# Gevab: a prototype genome variation analysis browsing server

**DOI:** 10.1186/1471-2105-10-S15-S3

**Published:** 2009-12-03

**Authors:** Woo-Yeon Kim, Sang-Yoon Kim, Tae-Hyung Kim, Sung-Min Ahn, Ha Na Byun, Deokhoon Kim, Dae-Soo Kim, Yong Seok Lee, Ho Ghang, Daeui Park, Byoung-Chul Kim, Chulhong Kim, Sunghoon Lee, Seong-Jin Kim, Jong Bhak

**Affiliations:** 1 Korean BioInformation Center (KOBIC), KRIBB, Daejeon, Korea; 2 Lee Gil Ya Cancer and Diabetes Institute, Gachon University of Medicine and Science, Incheon, Korea; 3 Department of Translational Medicine, Gachon University Gil Hospital, Incheon, Korea

## Abstract

**Background:**

The first Korean individual diploid genome sequence data (KOREF) was publicized in December 2008.

**Results:**

A Korean genome variation analysis and browsing server (Gevab) was constructed as a database and web server for the exploration and downloading of Korean personal genome(s). Information in the Gevab includes SNPs, short indels, and structural variation (SV) and comparison analysis between the NCBI human reference and the Korean genome(s). The user can find information on assembled consensus sequences, sequenced short reads, genetic variations, and relationships between genotype and phenotypes.

**Conclusion:**

This server is openly and publicly available online at http://koreagenome.org/en/ or directly http://gevab.org.

## Background

Most known genome browsers, such as NCBI genome [1] and Craig Venter's genome browsers [2], were built for consensus sequences from multiple individuals to construct a reference human genome. Examples of haplotype genome browsers are NCBI, UCSC [3], Ensembl [4], and Venter genome browsers. Recently, the first Asian (Chinese) diploid genome database was published, containing analysis and browsing facilities [5,6]. There are a number of general purpose genome annotation servers. They include Entrez Gene [7], Ensembl genes, OMIM [8] disease associations, HapMap [9], SNPedia [10], and genetic variations of several individual genomes such as Venter [11], Watson [12], YH (Chinese), and NA18507 (Yoruba) [13]. We have developed an individual genome variation analysis and browsing server (Gevab) for the first Korean personal genome sequence (KOREF).

This server is useful to analyze a diploid human genome produced to study the complex features of human genetic variations. The system integrated multiple variation information such as Venter, Watson, YH, dbSNP, and HapMap genotypes as well as gene information. Hence, users can comparatively study the genotypes in human. Gevab also provides information for SNPs, short indels, and SVs on the KOREF genome. Gevab has two parts: genome variation analysis and genome mapping.

## Materials and methods

### Data source

KOREF data were generated using the Illumina GA and resulted in 82.73 gigabase (Gb) of sequence (about 1248 million paired 36-base reads and about 504 million 75-base reads).

Using the MAQ (Mapping and Assembly with Qualities) [14] program, these sequences were aligned to the NCBI human genome reference (build 36, without Ns, 2,858,029,377 bp). In total, 99.9% of the NCBI reference genome was covered with an average of 25.92-fold depth (sequencing depth was 28.95-fold).

### Database and browser software

In the Gevab Korean genome variation browsing part, the consensus genome sequence and genetic variants include SNPs, short indels, and SVs can be displayed. Gevab used GBrowse [15] developed by GMOD [16] for variation viewing, and the genome map browser part was developed by KOBIC.

### Analysis of KOREF

From the KOREF genome sequence, 3.44 millions SNPs were identified and validated using Illumina 1 M-duo and Affy 6.0 BeadChip. We identified 342,965 short indels (-29 - +14 bp). Indels that co-occurred within a window size of 20 bp were filtered out, since they were primarily from length polymorphisms in homopolymeric tracts of A or T. Using paired-end reads, we found 2920 deletions and 415 inversion structural variants (SV) in the range of 0.1~100 kb. In addition, we detected 963 insertion events in the range of 175~250 bp. These insertions are present in the KOREF genome but absent in the NCBI reference genome. MySql and PHP, python, and AJAX were used in database construction and interface utility.

## Results

### Features of Gevab

The Gevab has genome variation analysis and genome map browser parts. The genome variation analysis part contains external public data sources, including the reference sequence of the human genome ((NCBI build 36), the Ensembl gene annotation, the Entrez gene annotations, dbSNP ver. 129 [17], OMIM annotations, and SNP frequencies of the HapMap population as well as genotype, indel, and structure variation of the KOREF. It is also integrated with other individual SNP variants such as James Watson's, Craig Venter's, and YangHuang's genotypes (Table 1). These external data sets are coordinated with the NCBI reference genome. A search can be done by putting in a genome location, a gene symbol, a RefSeq id, a dbSNP id, or an Ensembl gene id. When the user searches Gevab with a query, a graphical view of a chromosome ideogram and contigs are displayed. The gene locations within the 2 MB region centered on the query are also represented. For the displayed region in our browser, users can also download data with gff or fasta format ftp://ftp.kobic.re.kr/pub/KOBIC-KoreanGenome/.

**Table 1:** Features of Gevab, Venter, Watson, and YH genome browsers. Availability of features is indicated by "O" for "yes" and "X" for "no."

	Gevab	Venter	Watson	YH
**genome**	Korean	Caucasian	Caucasian	Chinese
**read mapping**	O	X	X	O
**sequencing coverage**	O	O	O	X
**genotype**	O	O	O	O
**indel**	O	O	X	X
**structure variation**	O	O	X	X

**variations to compare**	Venter, Watson, YH, dbSNP, HapMap	dbSNP	dbSNP, HapMap	dbSNP, HapMap

**web site**	http://gevab.org	http://huref.jcvi.org	http://jimwatsonsequence.cshl.edu	http://yh.genomics.org.cn/

### Gevab's map browser

The genome map browser provides reads mapping and quality information obtained from a personal genome project. A search can be done by chromosomal position. The width of a displayed region can be controlled. The browser also has zoom in and out and left and right movement functions.

**Figure 1:**
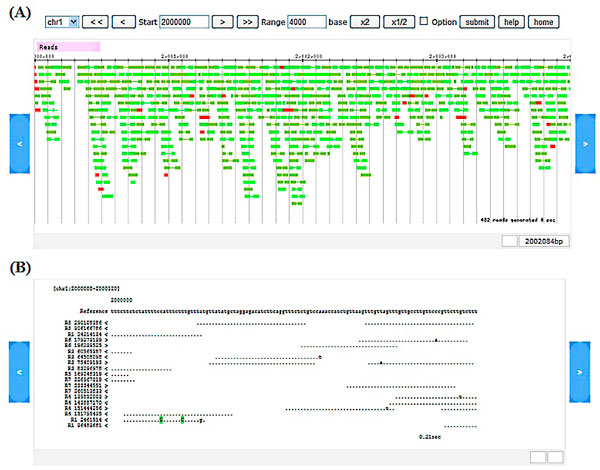
**A screenshot of the genome map browser**. (A) Graphic mode (B) Text mode.

When a user a chooses 1000 bp window size or longer, the browser shows a graphical view with forward and reverse pair-end reads in dark and light green, and single reads in red (Figure 1(A)). For shorter than 1000 bp window size, the browser is converted to a text mode that additionally shows quality information of mapped reads (Figure 1(B)).

### Gevab's variation browser

**Figure 2:**
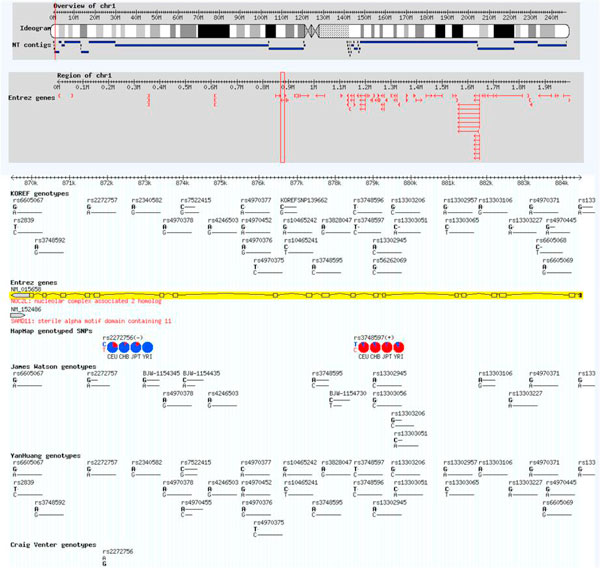
**A screenshot of the genome variation browser**.

To study genome variations, a genome variation browser is more useful than a genome map browser. As an example, if a user is interested in the "NOC2L" gene, s/he can get KOREF, Watson, YH, and Venter genome variation information through the variation browser part (Figure 2).

### KOREF data access

The KOREF database is developed and maintained by KOBIC (Korean Bioinformation Center). The database contains all the raw and processed data of KOREF, including KOREF consensus sequence, genetic variants, and short read alignments. These data are available for downloading. The KOREF data have been deposited in the NCBI Short Read Archive (Accession Number SRA008175).

## Conclusion

Gevab contains all the raw and processed data of a Korean genome sequence, variants, and annotation. Gevab provides open and public access to all data of an individual personal diploid genome.

The variation browser part was designed to present genetic variant evidence, including the position, number, and status of reads, GC content, and several mapping information. These provide valuable detailed information such as comparison and validation of genetic variations to further communities for sequencing individual genome.

## Competing interests

The authors declare that they have no competing interests.

## Authors' contributions

WYK developed the genetic variation browser and helped to write manuscript. SYK developed the genome map browser. THK wrote manuscript. SMA and DK provided the KOREF data. HNB, DSK, YSL, HG, DP, BCK, CK, and SL provided counseling on issues related to Gevab development. SJK supervised the whole project and guided to production of KOREF data. JB supervised the bioinformatic analysis and manuscript writing.

## Note

Other papers from the meeting have been published as part of *BMC Genomics *Volume 10 Supplement 3, 2009: Eighth International Conference on Bioinformatics (InCoB2009): Computational Biology, available online at http://www.biomedcentral.com/1471-2164/10?issue=S3.
